# Correlated mutations via regularized multinomial regression

**DOI:** 10.1186/1471-2105-12-444

**Published:** 2011-11-14

**Authors:** Janardanan Sreekumar, Cajo JF ter Braak, Roeland CHJ van Ham, Aalt DJ van Dijk

**Affiliations:** 1 Central Tuber Crops Research Institute, Thiruvananthapuram-695017, Kerala, India; 2 Biometris, Wageningen University and Research Centre, Box 100, 6700 AC Wageningen, The Netherlands; 3 Applied Bioinformatics, Plant Research International, Droevendaalsesteeg 1, 6708 PB Wageningen, The Netherlands; 4 Keygene N.V., P.O. Box 216, 6700 AE Wageningen, The Netherlands

## Abstract

**Background:**

In addition to sequence conservation, protein multiple sequence alignments contain evolutionary signal in the form of correlated variation among amino acid positions. This signal indicates positions in the sequence that influence each other, and can be applied for the prediction of intra- or intermolecular contacts. Although various approaches exist for the detection of such correlated mutations, in general these methods utilize only pairwise correlations. Hence, they tend to conflate direct and indirect dependencies.

**Results:**

We propose RMRCM, a method for Regularized Multinomial Regression in order to obtain Correlated Mutations from protein multiple sequence alignments. Importantly, our method is not restricted to pairwise (column-column) comparisons only, but takes into account the network nature of relationships between protein residues in order to predict residue-residue contacts. The use of regularization ensures that the number of predicted links between columns in the multiple sequence alignment remains limited, preventing overprediction. Using simulated datasets we analyzed the performance of our approach in predicting residue-residue contacts, and studied how it is influenced by various types of noise. For various biological datasets, validation with protein structure data indicates a good performance of the proposed algorithm for the prediction of residue-residue contacts, in comparison to previous results. RMRCM can also be applied to predict interactions (in addition to only predicting interaction sites or contact sites), as demonstrated by predicting PDZ-peptide interactions.

**Conclusions:**

A novel method is presented, which uses regularized multinomial regression in order to obtain correlated mutations from protein multiple sequence alignments.

**Availability:**

R-code of our implementation is available via http://www.ab.wur.nl/rmrcm

## Background

The amount of available sequence data is growing explosively. Annotation of those sequences at the protein residue level, which includes prediction of functional sites, binding sites and connections between sites, is essential in understanding structure and function of those sequences. Methods that associate specific parts of protein sequences with certain properties either use existing signatures or predict functional properties of the sequence *de novo*. Among the former, domain or motif based approaches compare sequences with databases of for example regular expressions, rule based motifs [1], or Hidden Markov Models [2]. Such signatures are inferred using sequence alignments, or information such as protein interaction data in combination with protein sequences [3,4].

Among methods that predict functional sites without using existing signatures, conservation of amino acids in sequence alignments is a well-known indicator of functional properties. In addition to conservation of columns, protein multiple sequence alignments often display correlations between columns. Such correlation contains information about which residues are located close to each other in 3D space and about functional sites [5,6]. Although several approaches to obtain such signals from sequence alignments exist [7-13], almost all of these are limited towards analysis of pairwise relationships between columns in the alignment. However, co-evolving contacts can be thought of as chains that percolate through the protein structure, inducing indirect dependencies [14]: when *m *and *n *are correlated, and *n *and *p *are correlated, *m *and *p *are likely to be detected as correlated as well, although in reality they do not directly influence each other. Hence, observed correlation does not necessarily imply that residues are located close to each other.

Several years ago it was proposed that graphical models, which take into account the network nature of dependencies, could be used to model protein structures [15]. At that time, the graphical model structure was learned with help from a protein structure and not from sequence data only. Only recently methods have appeared which, using sequence data only, analyze correlated mutations within the framework of graphical models [16-18].

We use recent advances in structure learning of graphical models (e.g. [19]) and learn a network structure where nodes describe columns in the multiple sequence alignment. This method can overcome the problem of indirect dependencies. In addition, because for every column in a multiple sequence alignment we predict (potentially) a number of other columns that are directly correlated with it, we find 'higher-order' multi-body contacts. This is relevant because it is known, based on contact statistics in protein structures, that multi-body contact frequencies are poorly predicted from pairwise contact potentials [20,21]. However, in the current study, the validation of our algorithm is focused on pairwise contacts because their assessment can be done in a straightforward way using protein structure data.

Our main contribution is a novel algorithm for correlated mutation analysis, Regularized Multinomial Regression based Correlated Mutations (RMRCM). We demonstrate its performance in network reconstruction using simulated datasets. The method was applied to analyze proteins and protein-protein interactions, and validated by comparing predicted residue connections with contacts observed in protein structures. We demonstrate the applicability of our approach by analyzing various types of datasets, and by predicting protein-peptide interactions.

## Methods

### Definition of the problem

Our objective was to identify correlations between columns in a protein multiple sequence alignment (MSA). These links contain information about which residues influence each other and can be used to predict which residues are located close to each other in 3D space. To achieve that, we defined a multinomial regression setup and fitted regression models where each column is regressed with all other columns in the MSA to find links between the columns. Our method RMRCM (Regularized Multinomial Regression based Correlated Mutations) was implemented in R and is available via http://www.ab.wur.nl/rmrcm.

### Multinomial regression setup

**Figure 1:**
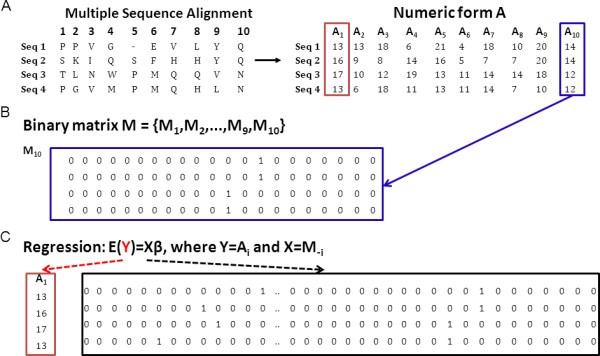
**Multinomial setup for correlated mutations**. **(A) **Mapping of amino acid characters to numerical factors. Matrix **A **represents the multiple sequence alignment, in which each amino acid is mapped to an integer (1-21). **(B) **Matrix **M **indicates the matrix to which **A **is converted: each column of **A **is expanded into 21 columns in **M**, as indicated for one particular column (column 10, A_10_, indicated by blue box). In this expansion, for each entry in the matrix **A**, the corresponding entry (14 and 12 in the example of column 10) in the M matrix is set to 1; the other 20 entries are set to 0. **(C) **Multinomial regression is used to find links between each column A_i _of **A **and all the columns in M_-i_, i.e. all columns in **M **except those representing A_i_. To do so, each column of **A **separately is used as dependent variable (Y) and all the columns in **M **that do not refer to that particular column of **A **are used as independent variables (X). In the example in this figure, Y = A_1 _(indicated with red box) and X = {M_2_,..,M_9_,M_10_}. Note that only part of X is shown.

In our method, an input MSA (**A) **is first converted to numerical form by mapping the sequences to factors with 21 levels (1 to 20 for aminoacids; gaps are mapped to 21). Subsequently, it is expanded to a matrix **M **with 21 times the number of columns of **A **by replacing each column by a binary matrix of 21 columns with a 1 representing the occurrence of each particular aminoacid (Figure 1). This matrix contains the starting data for RMRCM.

Consider the usual regression setup: we have a response variable **y **and a predictor matrix **X**, and we approximate the regression function by E(**y**) = **Xβ **where **β **are the regression coefficients. Our aim is to fit a regression model for each column of the MSA, and hence, the factor representing column i from **A **is taken as **y**. As **X**, we take the matrix **M**_-i_, with **M**_-i _the matrix **M **after deleting the 21 columns that refer to column i in **A**. Hence, in our regression problem for column i of the MSA, Y equals **A_i _**and we find a model which explains as much as possible variation in **A_i _**using the independent variables in X = **M_-i_**. This is repeated for each column i in **A **separately. As **y **is a factor with 21 classes, the regression model is generalized to that of multinomial regression, in which we take the symmetric form proposed by ref. [22]. After each fit, the coefficients **β **describe the relationships between columns in **M_-i _**with the i^th ^column in **A**. These are then projected back to describe relationships of columns in **A **with each other. For this we use the sum of the absolute values of the regression coefficients. This results in links being predicted between various columns of the MSA.

Because the problem contains many parameters and a relatively small number of datapoints, regression would in general result in many links being predicted. Lasso [23] is a popular method for regression that uses an L_1 _penalty to achieve a sparse solution, ridge regression similarly uses L_2_, and the elastic net regression method is a compromise between lasso and ridge regression [24]. We fit the multinomial regression models with elastic-net penalties using the algorithm implemented in the R-package glmnet version 1.4 [22].

The elastic net solves the following problem (equation 1):

(1) $$\underset{\beta }{\arg\max}[l(y,X,\beta )-\lambda P_{\alpha }(\beta )]$$

with *l*(**y**, **X**, *β*) the log-likelihood of the multinomial regression model [22] which depends on the data **y**, the predictor matrix **X **and the coefficients *β*. *P_α _*is the elastic-net penalty, which is a compromise between the ridge regression penalty (α = 0) and the lasso penalty (α = 1):

(2) $$P_{\alpha }\left(\beta \right)=\overset{p}{\underset{j=1}{\sum }}\left(1-\alpha \right)\frac{1}{2}\beta_{j}^{2}+\alpha |\beta_{j}|$$

with *p *the number of coefficients in *β*. Non-zero values for *β *indicate predicted links between the y-column and a subset of the X-columns. The regularization parameter λ determines the strength of the elastic net penalty and the higher value it has, the less coefficients will be non-zero. Note that the coefficient for the i-j pair of columns in **A **can be different from that of j-i; hence, the coefficients were symmetrized by taking the average.

### Selecting the tuning parameters

After some preliminary testing, α was set to 0.99 in all experiments presented in this paper. The links are computed for an entire path of solutions of the regularization parameter λ. The default sequence of 100 values of λ was used, and for selecting the best λ, we tested using the Bayesian Information Criterion (BIC) [25]. BIC is computed as BIC = -2 *l*(**y**, **X**, *β*) + k ln(n) where *l*(.) is the log-likelihood with *β *the solution to Eq. (1) for given λ, k is the number of parameters and n is the number of data points. We chose the predicted links with minimum BIC for each column separately, i.e. the regularization parameter was chosen independently for each column. In addition to the BIC-based selection of an optimal value of λ, we also tested using the sum of the coefficients *β *obtained over all values of λ. Because this gave always at least comparable and often somewhat better results than using the BIC-based selection, unless otherwise mentioned reported results were obtained using the sum-of-coefficients approach.

### Artificial datasets

To test RMRCM, we used sequence alignments derived from artificial networks. These networks were generated as follows. Using 200 nodes, an edge density of 0.1, 0.25 or 0.5 was used and interactions between nodes were randomly chosen with this probability. Here, edge density was defined as the fraction of edges out of the total possible number of edges (200*200*0.5-0.5*200 = 19,900); hence, the networks contained approximately 1990, 4975 or 9950 edges, respectively. For each edge density value, three different replicate networks were generated. For each network, different alignments were generated using Markov Random Field potentials [26,27] which determine a probability for each amino acid (20 amino acids + 1 gap for a total of 21 characters) at each node of the network, generating an alignment with 200 columns. These potentials consisted of node and edge terms to encode preferences for specific amino acids at positions in the alignment, and interactions between positions, respectively. For each node, randomly one amino acid was selected as most preferred, with associated node potential value p_prefnode _(0.1 or 0.3); all the other amino acids at this node had a similar potential of (1-p_prefnode_)/20 (such that the total probability adds up to 1). In this way, preference for a certain amino acid at each position was encoded, whereas the probability for all other amino acids is equal. For the edge potentials, sets of preferred amino acid pairs were generated for each edge separately. For each of the amino acids, two amino acids were chosen randomly as the preferred partners of that amino acid. Preferred associations had edge potential value p_prefedge _(0.1 or 0.3; "weak" and "strong" interaction, respectively); others had potential value of (1-p_prefedge_)/(21*21-21* 2) (such that the total probability adds up to 1). With these edge potentials, preference for certain amino acid combinations at certain pairs of positions was encoded.

Subsequently, Gibbs sampling was used to generate samples, i.e. sequences in the alignment. The number of iterations was set to 100,000, and every 50^th ^iteration the node labels were recorded. Three different sequence alignments were generated from this, using the last 50 samples, the last 500 samples or the last 1,000 samples, with 50 iterations in between. Here each sample constitutes one labelling of the network, i.e. it represents one sequence in the sequence alignment. Hence, N_seq_, the number of sequences in the samples, was 50, 500 or 1,000. In total, 108 sequence alignments were generated with the combination of three edge densities, two levels of p_prefnode_, two levels of p_prefedge_, three values of the number of sequences in the sample and each combination replicated three times.

The values of the node and edge potentials as defined above were chosen after some initial tests such as to generate a range of different sequence similarities within alignments, as well as a range of interaction strengths, comparable to what was observed in biological alignments (for which we used PFAM entries, see below). In those alignments, the average sequence pairwise sequence identity was 0.34 +/- 0.14. In our case, the sequences generated using the highest values for preferred node and edge potentials also obtained a pairwise sequence identity of 0.34 (+/- 0.23); those with lower values for the potentials obtained somewhat lower values (0.15 +/- 0.20). In order to calculate and compare interaction strengths between the artificial aligments and biological alignments, we used mutual information (MI, see below) calculated for all pairs of columns in the sequence alignment. In the biological alignments, the average value of MI was 0.14 +/- 0.08. In the artificial alignments, the set with p_prefnode _= 0.1 and p_prefedge _= 0.3 obtain a quite comparable value of MI: 0.11 +/- 0.05. The cases with other values for p_prefnode _and p_prefedge _obtained somewhat lower values of MI (0.09 +/- 0.02).

To analyze the influence of correlations between the samples, instead of sampling every 50 iterations we also tested sampling every 200 iterations, as well as sampling every iteration. The latter might correspond to the biological situation of having sequences that are relatively closely related phylogenetically. To simulate the situation of having even more closely related sequences in part of the dataset, we tested adding additional copies of a given sequence to the data in order to analyze the impact of these on the performance of the method.

In addition, two different datasets of alignments with noise were generated. In the first set ("position-noise"), a certain percentage (10% or 25%) of all positions in the sequence alignment were randomly changed into another amino acid. This type of noise is a crude way to simulate misalignment. In the second set ("sequence-noise") new sets of sequences were generated using only node potentials and without edge potentials (hence positions are not coupled to each other). Next, these sequences were combined with the original sequence alignments, such that 10% or 25% of the original sequences were replaced by these newly generated sequences. This second type of noise simulates the situation that sequences which are included in an alignment do not originate from proteins with the same set of interactions between residues; this might in particular happen in the case of analysis of interacting proteins where ortholog pairs from various species are added to the alignment, and where *a priori *it is unclear if all of these do indeed interact as is the case for the 'seed' pair of proteins.

### Validation of the predicted links on simulated datasets

To assess the performance of RMRCM on the above-mentioned simulated datasets, we calculated the area under the Receiver Operator Characteristic (ROC) curve for our predictions, using the R-package ROCR [28]. As a first approach, we selected the predicted links corresponding to the minimum BIC value (for each MSA column separately), and for computation of AUC we used the absolute values of the coefficients (β-values) as quantitative score. As a second approach, we used the sum of the absolute values of the coefficients summed over the whole regularization path as a score. As a third approach, we also tested using the number of models (out of 100 models for different λ values) in which a particular link is present. Because this approach gave very similar results to using the sum over the whole regularization path we do not report these results here. Because using the minimum BIC resulted in lower AUC values than using the sum of the coefficients over the whole regularization path, only results for the latter approach are reported for the artificial datasets. As a standard method to compare the results of our method for the simulated datasets mutual information (MI) was used to predict links [8]. MI was calculated including the correction using mutual entropy proposed previously [8]. We tested that this indeed gives better performance compared to uncorrected MI but we show only results for corrected MI.

### Biological datasets

As a standard benchmark for contact prediction we analyzed the contact prediction cases from the most recent CASP experiment (CASP9), for which both target sequences as well as predictions submitted by CASP participants were obtained via http://www.predictioncenter.org. Sequences to generate a multiple sequence alignment for each target sequence were obtained with blastp against the NR dataset with an E-value cutoff of 0.01. Because CASP9 results have not been published yet, we calculated the prediction performance of CASP9 cases using the raw predictions that we obtained via http://www.predictioncenter.org. We checked they are consistent with results described in http://predictioncenter.org/casp9/doc/presentations/CASP9_RR.pdf. As described in the CASP8 contact prediction assessment paper [29] evaluation was performed on FM and TBM/FM domains (i.e. cases for which no homologous structures were available), which in CASP9 constituted in total 28 domains. Residues were considered to be in contact if their Cβ atoms (Cα for glycines) were within a distance of 8 Å. For target domains of length *L*, the top ranked *L*/5 and *L*/10 predictions according to the predictor scores were evaluated, and only contacts for residues separated at least 24 residues along the sequence were taken into account. Predictions were evaluated using two different scores, accuracy [TP/(TP+FP), where TP = true positives and FP = false positives], and *Xd*, which measures how the distribution of distances for predicted contact pairs differs from the distribution of all pairs of residues in the target domain structure [30].

As a larger benchmark, we obtained a set of sequence alignments related to PFAM entries [31]. In order to limit the computational requirements for this analysis, we restricted this analysis to PFAM entries having exactly one match of at least length 50 residues to a representative PDB structure. We separately analzyed cases with 200-500 sequences in the alignment, 500-1000, 1000-2000 or 2000-4000 sequences. The number of cases in those four categories were 604, 356, 62, and 234, respectively. The performance of RMRCM on these cases was analyzed using the CASP criteria.

In addition to these two benchmarks, we analyzed various biological datasets (Table 1), including both intra- and intermolecular analyses, which were previously analyzed using various methods. In order to compare our results, we used validation criteria as described in the original publications; this means that the exact setup varies somewhat between the different cases. In particular, for the response regulator and the SK-RR datasets, a cutoff value of 6 Å was used to define short distances, for the MADS domain proteins 5 Å, and for the CDD and PDZ-peptide sets both 5 Å and 15 Å.

**Table 1:** Biological datasets used for RMRCM performance asssessment

Dataset	N_sets_^a^	N_prot_^b ^	N_col_^b^	Structure^c ^	Reference
**PFAM**	1256	200/3975	10/521	Several^d^	[31]
**CASP9**	28	5/501	21/227	Several^e^	www.predictioncenter.org
**MADS**	12	34/339	78/218	1n6j	[36]
**Response regulators**	1	1433	186	1xhe	[16]
**CDD**	36	125/1922	34/411	Several^f^	[8]
**SK-RR**	1	4934	184	2c2a, 1pey, 1f51	[16]
**PDZ-peptide**	1	2385	162	1n7f	[33]

^a ^N_sets_, number of separate multiple sequence alignments. Four datasets consists of several multiple sequence alignments, each of which is analyzed separately. For these, the number of proteins and the number of columns mentioned are the minimum and maximum found in these sets.

^b ^N_prot_, number of proteins; N_col_, number of columns in the multiple sequence alignment.

^c ^PDB identifier of structure used to compare predicted residue contacts.

^d ^Obtained via PFAM.

^e ^Obtained via www.predictioncenter.org.

^f ^See ref. [8].

To align the sequences, MUSCLE [32] was used. To compare residue-residue contacts predicted by RMRCM, structure data were used (Table 1). Although we treat gaps in our approach on equal footing with amino acids, biologically it does not make sense to analyze columns with many gaps. For that reason, we used a cutoff on the number of gaps in a column, which was set to 50%; columns with more gaps than the cutoff were excluded from all analyses.

The PDZ-peptide dataset consists of human and *C. elegans *PDZ domains with associated binding peptide sequences for each of them. For this dataset, in addition to predicting contacts between the protein and the peptide, the model learned by RMRCM based on the human data was also used to predict interactions between *C. elegans *PDZ-peptide pairs. For all *C. elegans *PDZ-peptide sequences, after aligning with the human sequences, the log likelihood was calculated by summing the log likelihood for each position (*l *in equation 1), using the RMRCM model selected based on minimum BIC (including a pseudocount of 1.0/210 in the likelihood calculation). In this way, the *C. elegans *interacting PDZ-peptide pairs obtain a score based on the model trained with human data only. In order to compare the scores for interacting pairs with those for non-interacting pairs, a set of non-interacting PDZ-peptide pairs was generated. To do so, the data for *C. elegans *were randomized such that peptides were randomly assigned to PDZ domains for which no interaction was observed with that peptide; note that this means that our non-binding dataset might contain a subset of PDZ-peptide pairs that do interact and hence the reported performance might underestimate the real performance. In total, in addition to the 1199 experimentally observed interacting PDZ-peptide pairs, 1199 non-interacting pairs were assembled. To assess the dependence of the interaction prediction on the similarity between *C. elegans *and human PDZ sequences, we calculated binding site identity as described previously [33].

## Results

Indirect dependencies between columns in a multiple sequence alignment (MSA) cannot easily be distinguished from direct dependencies by currently available pairwise methods for correlated mutation detection. This limits the applicability of such methods for the prediction of binding sites or residue-residue contacts. To deal with this, we use recently developed methods in structure learning of graphical models which apply regularization in order to learn sparse network structures, i.e. filter out indirect dependencies. To do so, we frame the problem of finding correlated mutations between columns in a protein multiple sequence alignment in a multinomial regression setup. We convert each column in the MSA into 21 different columns (20 amino acids + gap) with 1 (0) in each column indicating presence (absence) of that amino acid in each particular sequence (Figure 1). Subsequently, the resulting binary matrix is used as independent variables ("X") and each column in the original MSA on its turn is used as dependent variable ("Y") in a regression approach; in doing so, we find a model for each column in the original MSA which explains as much as possible of its variation using the information from all other columns in the binary matrix. Regularized regression allows fitting models to such large datasets and comprises a penalty parameter to get a balance between a good fit and a small number of coefficients. These coefficients describe the resulting predicted links between columns in the MSA. To select the optimal penalty parameter, we apply the Bayesian Information Criterium (BIC), or a sum of coefficients found at different values of the penalty parameter. The resulting approach is named Regularized Multinomial Regression based Correlated Mutations (RMRCM); more details are presented in the Methods section. In principle, our method finds 'multi-body' contacts between each residue which is used as dependent variable and all residues for which non-zero coefficients are found with that residue. However, we simply use the non-zero coefficients here as predictors for pair-wise interactions between residues.

To obtain insight into the performance of our algorithm, as well as the influence of factors such as noise or sequence similarity, we analyzed several simulated datasets. We compared the performance of our approach with the often used mutual information (MI) approach for correlated mutations. Next, a number of protein sequence alignments were analyzed for which the predicted contacts were validated with protein structure data and where the performance of RMRCM was compared with MI as well as some other approaches. In this step of validation and application to biological datasets we used both standard benchmark sets (CASP, PFAM) as well as a number of datasets which have been previously analysed.

### Validation: artificial datasets

Artificial sequence datasets were generated based on various artificial networks with different interaction densities, using Gibbs sampling with a potential function defined over nodes and over edges between interacting nodes, where interactions could be either weak or strong. In addition to the generated sequences, datasets with added noise were also analyzed (see Methods for description).

The performance of MI and RMRCM was assessed by comparing the predicted interaction strengths between pairs of columns in the MSA with the known network structure using the Area Under the Curve (AUC) value. We first discuss the results for MI. For the datasets without noise, on the alignments with only 50 sequences, or from networks with edge density 0.5, MI resulted in performance very close to random performance. For datasets with 500 or 1,000 sequences, the performance of MI mainly depended on the edge potential (all cases with weak interactions between nodes had random performance) and on the interaction density (for the cases with strong interactions between nodes, the performance was better with lower interaction density). In addition, increasing the number of sequences from 500 to 1,000 improved the performance (although in most cases only slightly).

For the datasets with 10% "position-noise", the performance of MI as predictor seemed hardly affected, but with 25% noise there was in most cases a clear effect. For the datasets with "sequence-noise", the noise had clear impact on the performance of MI predictions at 10% noise added.

**Figure 2:**
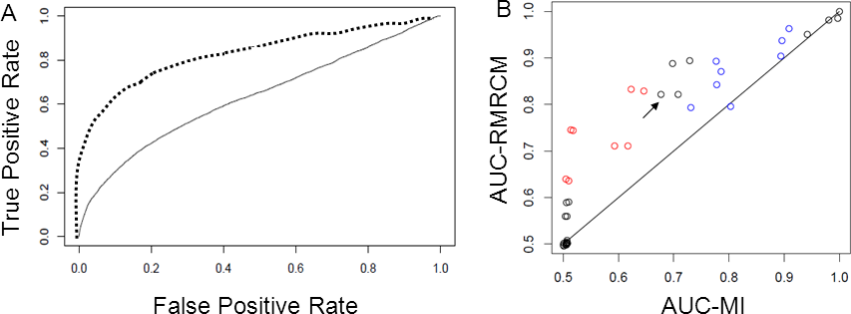
**Performance of RMRCM compared to performance of MI on artificial datasets**. **(A) **Representative example of ROC curves for RMRCM (dashed) and MI (continuous line) for simulated dataset with network edge density 0.25, and 500 sequences in the multiple sequence alignment. Performance is assessed by comparing predicted contacts with those in the network used to generate the artificial sequences. **(B) **AUC values for MI (x-axis) vs. RMRCM (y-axis) for datasets with 500 or 1,000 sequences. Arrow indicates particular case illustrated in panel A. Black indicates data without noise, red with "position-noise" (10% and 25%) for interaction density 0.25 and blue with "sequence-noise" (10% and 25%) for interaction density 0.1.

In almost all cases, RMRCM resulted in higher AUC (better prediction performance) than MI (Figure 2). This held true in particular at the intermediate edge density of 0.25, where MI predictions deteriorated compared to edge density of 0.1 whereas our approach suffered much less. For example, when using 500 sequences at edge density 0.25, the AUC value for MI was 0.68 +/- 0.02 for the case of weak node potential values and strong interactions between columns; RMRCM obtained an AUC of 0.82 +/- 0.01. The datasets resulting from sampling using weak edge potential still obtained random AUC scores using RMRCM, probably indicating the absence of any detectable signal in these cases. For the datasets with noise added, again RMRCM results were better than MI results (Figure 2).

A general issue in correlated mutation analysis is that when sequence similarity is high (sequences originate from closely related species), it can be difficult to disentangle correlation and conservation [34]. To investigate this, we analyzed the effect of sampling frequency when generating the datasets. When using 200 iterations instead of 50 as used above as interval for writing output during the Gibbs sampling (and hence generating sequences that are somewhat less similar), the performance only slightly improved (for both MI and RMRCM). However, when using highly correlated samples (sampling every iteration) there was a clear impact on performance, which dropped considerably. For example, for the same networks with density 0.25 mentioned above, AUC for MI was 0.59 +/- 0.01 and that for RMRCM was 0.63 +/- 0.02.

As the most extreme limit of high correlation between a number of sequences in the alignment, we tested the influence of adding additional copies of a given sequence to the alignment (using the sets with 500 sequences and adding 10, 25 or 50 copies of a randomly chosen sequence). For MI, there was a clear effect; for example, for networks with density 0.1, strong edge preferences and weak node preferences, the AUC for MI with 10 added copies was 0.98 +/- 0.002, with 25 copies 0.95 +/- 0.01 and with 50 copies 0.92 +/- 0.01. However, for RMRCM, there was hardly any change in performance (data not shown) meaning that also in this respect it performed better than MI.

### Protein datasets: intramolecular analysis

To test our method on biological datasets and demonstrate its applicability, we first tested two standard benchmarks, *viz*. CASP contact prediction cases and a large set of PFAM entries. In addition, we chose to analyse various datasets that have been analyzed previously and where prediction performance was assessed using available crystal structures. In order to be able to compare with those previously obtained results, we used validation criteria as described in the original publications. This means that the exact setup varies somewhat between the different cases, but it has the important advantage of allowing comparison with results obtained by developers of various methods, who are experts on those methods and would be expected to obtain the best result possible with their respective methods.

### CASP9 contact prediction

**Figure 3:**
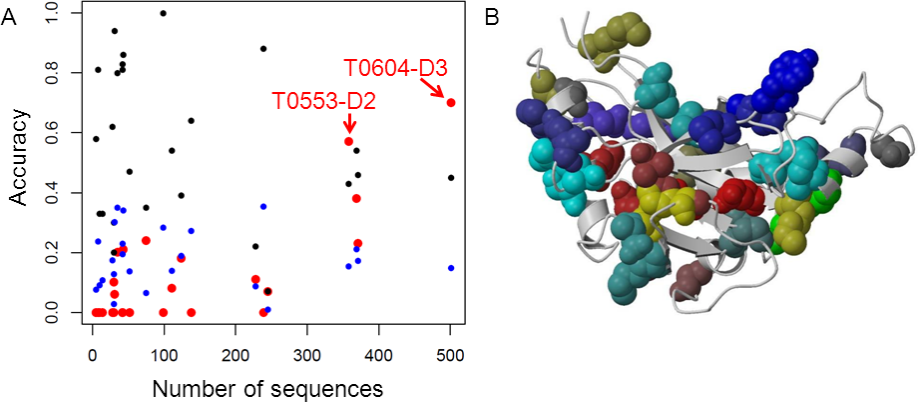
**CASP9 prediction performance**. **(A) **CASP prediction performance as measured by accuracy for the L/10 contacts (L = length of target sequence) with the highest predicted scores, as a function of the number of sequences in the sequence alignment. Red, RMRCM prediction performance; black, best prediction performance among all CASP participants; blue, average prediction performance among all CASP participants. For two out of the four cases with the highest number of sequences, RMRCM performance is better than that of any of the CASP participants; for these two cases, labels are added to identify the CASP targets. **(B) **Protein structure for target T0604-D3 in cartoon representation, together with the 20 top-ranked predicted contacts in spacefill (20 corresponds to L/10 for this protein). Identical colors for the residues indicate pairs of residues for which a contact was predicted. Out of those 20 residues, 14 are contacts according to the CASP criteria, and most of the others are relatively close to each other as well (within 15 Å).

Contact prediction target cases were obtained from the latest CASP round. We observed a clear dependence of RMRCM performance for contact prediction on the number of sequences in the alignment (Figure 3A). We also provide the average CASP performance in Figure 3 as additional comparison. In fact, for two of the four alignments with more than 300 sequences (T0604-D3, 501 sequences, and T0553-D2, 358 sequences), the performance of RMRCM was better than that of any of the CASP9 participants, based on accuracy, i.e. the fraction of predicted contacts that are indeed contacts in the crystal structure. When based on Xd, which measures how the distribution of distances for predicted contact pairs differs from the distribution of all pairs of residues in the target domain structure, this was only the case for T0604-D3, the one with the highest number of sequences (Figure 3B). For all four of these alignments RMRCM performance was better than the average CASP performance. For many of the cases for which only a small number of sequences was available, RMRCM performance was not good, even not in comparison to the average performance of CASP participants. Note however that various machine learning algorithms are used in CASP, which incorporate various features and as such the performance of RMRCM as a contact predictor could be boosted by combining the correlated mutation search with such approaches. This could include for example specialized beta-sheet contact prediction algorithms such as applied by NNcon [35].

The dependence of RMRCM performance on the number of sequences in the input alignment as observed with the CASP datasets, was similar to what was observed for the artificial datasets (see above). Another observation from the artificial datasets, as mentioned above, was that the contact density had a large influence. However, we did not find such influence for the CASP cases although there is indeed quite some variation in contact density for the CASP cases (data not shown).

### PFAM

We selected a subset of PFAM entries (see Methods for criteria), which we subdivided according to the number of sequences in the alignment. A clear dependence for the contact prediction performance was observed on the number of sequences in the alignment, such that going from less than 500 sequences via less than 1,000 sequences to between 1,000 and 2,000 sequences the performance clearly improved (Table 2). Increasing the number of sequences even further (between 2,000 and 4,000) did not give any additional improvement in performance. Although direct comparison with performance on the CASP cases is obviously not possible, it is reassuring that the average performance measures observed with the cases with at least 1,000 sequences would place RMRCM among the best performing approaches when compared with available CASP prediction results. Performance of Mutual Information on all those datasets was clearly worse; for example, for the sets with between 1,000 and 2,000 sequences, the accuracy for the L/10 best scoring contacts with MI was only 0.18 +/- 0.17 compared with 0.23 +/- 0.24 for RMRCM.

**Table 2:** Contact prediction performance on PFAM datasets

N_prot_^a^	Accuracy^b^		Xd^c^	
	**L/5^d^**	**L/10^d^**	**L/5^d^**	**L/10^d^**

**200-500**	0.10 (0.09)	0.11 (0.12)	4.1 (4.2)	4.6 (5.5)
**500-1000**	0.16 (0.14)	0.21 (0.18)	6.3 (5.2)	8.0 (6.9)
**1000-2000**	0.24 (0.18)	0.32 (0.24)	9.3 (7.5)	12.1 (9.3)
**2000-4000**	0.25 (0.19)	0.33 (0.26)	8.8 (7.9)	11.8 (10.1)

^a ^N_prot_, number of protein sequences in the alignment.

^b^Accuracy, fraction of predicted contacts that is correct according to the crystal structure. Contacts are defined according to the CASP criteria (Cβ atoms (Cα for glycines) within a distance of 8 Å; only contacts for residues separated at least 24 residues along the sequence were taken into account).

^c ^Xd, measures how the distribution of distances for predicted contact pairs differs from the distribution of all pairs of residues in the target domain structure.

^d ^Highest ranked predicted contacts were assessed, using either L/5 or L/10 contacts. Here L refers to the length of the target sequence. Values for accuracy and Xd are averages (standard deviations).

Because these datasets represent a typical setting in which RMRCM could be applied, we also analyzed the running time. On average, for the sets with 2000 - 4000 sequences RMRCM needed approximately 15 hours on a single CPU for a single dataset, meaning that using any reasonable sized compute cluster one can analyze fairly large amounts of data. For the smaller sets the running time was much smaller, for example for the sets with 500-1000 sequences it was on average less than 2 hours. Running time showed a positive Pearson correlation coefficient of ~0.45 (p~10^-12^) with both number of columns in the alignment and number of sequences in the alignment.

### MADS domain proteins

**Figure 4:**
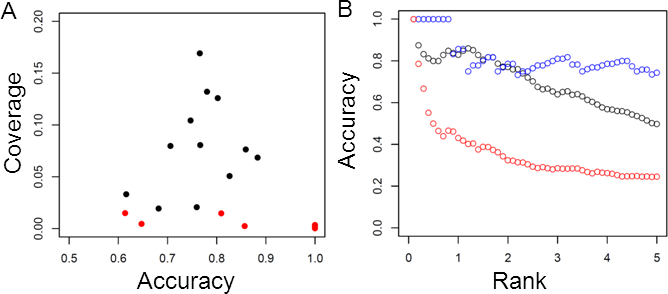
**Contact prediction performance**. **(A) **MADS domain proteins: accuracy (TP/(TP+FP)) and coverage (TP/(TP+FN)) for prediction of residue contacts using RMRCM (black), and CAPS, a method we applied previously to this dataset (red). Note that for CAPS, in three cases no links were predicted at all; these cases are not shown. **(B) **Response regulator proteins: accuracy vs. rank percentile for predicting contacts for mutual information (red), RMRCM (black) or RMRCM restricted to 60 positions among which maximum MI was found (blue).

The MADS domain protein dataset consists of 12 Arabidopsis MADS domain proteins with homologous sequences from various plant genomes which we previously analysed using CAPS, an algorithm which uses BLOSUM and calculates Pearson correlation coefficients between the transition probability scores (between pairs of sequences) observed in one column and each other column [7,36]. For these proteins, the RMRCM predictions in almost all cases had a significant overrepresentation of short distances compared to the crystal structure: using a χ^2^-test, all but two out of twelve MADS datasets had p-values below 0.05, and in most cases the p-value was much smaller; the average for the ten cases with p < 0.05 was 0.006 +/- 0.01. Although the distance enrichment of the results previously obtained with CAPS was in some cases slightly better than for RMRCM, the number of predicted links was much higher with our new approach (Figure 4A). To make a proper comparison, we calculated the F-scores [28] for the predicted links. With 0.19 +/- 0.09 this was much higher for RMRCM than what was previously obtained with CAPS (0.012 +/- 0.015). We also used MI on those datasets, and found that the distance enrichment of MI-predicted links was much worse than what was obtained with CAPS or RMRCM (data not shown).

Note that there is quite some variation in the performance for the various MADS domain proteins, which is mainly related to the different amount of sequences in the multiple sequence alignments for those proteins, as observed already when using CAPS (see [36]) and in line with results mentioned above.

### Response regulator proteins

For the response regulator proteins, we calculated the accuracy vs. rank percentile for predicting contacts (Figure 4B) as was previously done by Weigt *et al*. [16]. Here, the predicted contacts were sorted based on the score assigned by RMRCM, and the accuracy of the top *n*% predicted contacts, i.e. the fraction of predicted pairings which indeed were in contact in the structure, was calculated, for various values of *n *(0-5%). Figure 4B can be directly compared with Supporting Information Figure S7 in Weigt *et al*. The performance of our procedure is quite comparable to their performance, which is remarkable because our approach is computationally much less expensive. Weigt *et al*. had to restrict their analysis to a subset of only 60 positions in order for the problem to be computationally tractable whereas we can easily analyze more columns (for this particular dataset, 187 columns were analyzed with one CPU within a couple of hours).

### CDD

As a final set of protein MSAs for intramolecular analysis we used data previously analyzed using MI [8], assembled using the Conserved Domain Database (CDD). Here, combining results for all different datasets, in the crystal structures 6% of the residue pairs had distance below 5 Å and 35% below 15 Å. For the BIC-based predictions, these percentages were 25% and 67%, respectively; this increased to 41% and 73% when restricting to the ten highest scoring pairs for each dataset. The performance when using the sum of the coefficients along the whole regularization path was quite comparable although slightly worse than when using BIC. When restricting to the pairs that had overlap with the top 100 mutual information-based pairs, these percentages were somewhat higher (45% and 79%). The top 100 mutual information based pairs had somewhat lower enrichment with 24% and 66% within 5 Å and 15 Å, respectively.

Comparison with the results obtained by Martin *et al*., who analyzed only pairs of residues that have no additional partners ("isolated pairs"), indicated that in our case the distance enrichment was slightly lower, but again the number of predicted links was much higher. For example, for the alignments with at least 150 sequences, using the top 10 predictions for each dataset, we obtained a fraction of predicted residue pairs within 5 Å of 0.44 compared to 0.66 for Martin *et al*.; however, in their case, only 32 pairs were predicted, compared to 240 in our case.

## Protein datasets: intermolecular analysis

### SK-RR

For the SK - RR interacting proteins, we tested allowing either only intermolecular contacts, or both inter- and intramolecular contacts (the intramolecular contacts were not further analyzed but in building the prediction model they can influence the intermolecular contacts). Based on comparison with intermolecular contacts from the available crystal structure, the exact setup did not influence much the results. Weigt *et al*. previously predicted 6 links which indeed all had a short distance in the crystal structure. When taking the 6 links with the highest sum of coefficients based on minimum BIC or using the sum of all coefficients along the regularization path, in most cases we also predicted only pairs that were indeed in contact (except in the case of using only intermolecular contacts and using minimum BIC; in that case, one of the predicted links had a larger distance). Most of the predicted links were the same as found by Weigt *et al*.

### PDZ-peptide

The PDZ-peptide dataset consists of a set of human PDZ domains and associated binding peptides for each PDZ, and a similar set of *C*. elegans PDZ domains and interacting peptides. For this dataset, we used RMRCM to predict residue contacts between the PDZ domain and the peptides, but in addition we tested using RMRCM for predicting which PDZ domain interacts with which peptide. First, we predicted PDZ-peptide residue connections. Here, using only intermolecular contacts resulted in much worse intermolecular contact prediction than using both intra- and intermolecular contacts. We compared the top 50 predicted intermolecular links obtained with the model using both inter- and intramolecular contacts for RMRCM with those predicted by MI. Both had about an equal number of predicted contacts which were found in the crystal structure within 5 Å (10%). However, the number of predicted contacts found within 15 Å in the crystal structure was much higher for RMRCM (80%) compared to MI (50%); the value found for MI is equal to the overall percentage of pairs of residues found within 15 Å of each other in the crystal structure. Hence, although MI does not improve over a random prediction, RMRCM clearly does.

**Figure 5:**
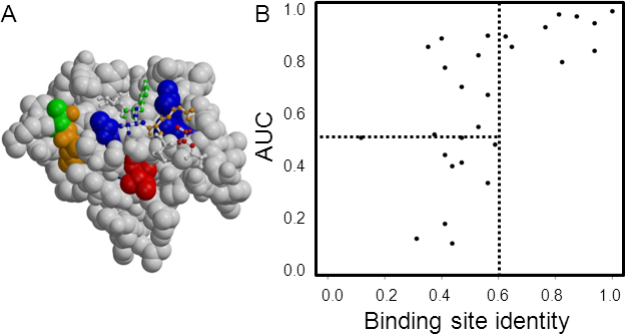
**PDZ-peptide contact and interaction prediction**. **(A) **Residues predicted on PDZ domain are mostly located in the peptide binding site. Spacefill indicates PDZ domain and colored spacefill indicates PDZ residues predicted by RMRCM to interact with the peptide; ball-and-stick indicates peptide that interacts with the PDZ domain. Colors on the peptide indicate with which residues with corresponding colors on the PDZ domain those residues are predicted to connect; in one case, a residue on the PDZ domain obtains two colors (green and orange) because connections are predicted with two peptide residues. In some cases (e.g., blue residues) the predicted connections are between residues which are contacting each other; in other cases (e.g., orange residues) this is not the case, although the residues predicted on the PDZ domain are still relatively close to the peptide. Two additionally predicted residues on PDZ are at the backside of the molecule and are not visible. **(B) **Prediction of *C. elegans *PDZ-peptide interactions using human interaction data as training set. AUC values for *C. elegans *PDZ-peptide interaction prediction based on human interaction data (y-axis) vs. binding site identity of the *C. elegans *PDZ sequence with the best-matching human sequence (x-axis). Lines indicate randomly expected AUC (0.5) and binding site identity above which good prediction performance is obtained (0.6).

Indeed, the majority of the residues predicted using RMRCM on the PDZ protein are in close proximity of the ligand peptide (Figure 5A). In some cases, the residues on the PDZ protein also contact with the residue on the ligand with which a connection is predicted whereas in other cases the residues found on the PDZ domain just are near the binding site but do not directly contact the peptide residue with which a connection is predicted (Figure 5A).

In addition, we used the model trained with human data to predict interactions for *C. elegans *data based on the likelihood score. When using a model trained using both intra- and intermolecular links, this resulted in poor differentiation between interacting and non-interacting *C. elegans *PDZ-peptide pairs. However, when using a model trained using only intermolecular links, there was a clear differentiation; for example, among the top 100 PDZ-peptide pairs with the highest score, 97 were indeed interacting, whereas among the lowest 100 scores, 73 were indeed non-interacting.

Previously, a simple method was proposed using binding site similarity between PDZ domains to predict interactions with peptides [33]. In line with the results of that method, we observed a relationship between the maximum binding site identity of a *C. elegans *PDZ with the human PDZ sequences, and the AUC we obtained for prediction of interactions of that *C. elegans *PDZ sequence (Figure 5B). Interestingly, only below a binding site identity of 0.6 the interaction prediction became less reliable. This is an improvement over the previously observed binding site identity which was needed to reliably transfer interaction information between PDZ domains (where a value of 0.7 distinguished PDZ domains with similar from PDZ domains with distinct binding profiles) [33]. Hence, at least for this particular dataset, RMRCM is able to push the limit of cross-species sequence-based interaction prediction towards lower similarity levels. Note however that we provide here just one example of using RMRCM as a way to predict interactions, because our focus is on predicting residue-residue connections. Further work would be needed to assess the performance of RMRCM as a general protein-protein (or protein-peptide) interaction predictor.

## Discussion

We present RMRCM, a method for correlated mutation analysis using regularized multinomial regression, and demonstrate its performance and applicability with various datasets. Even though correlations between columns in a multiple sequence alignment can arise due to various factors, we focus here on using our algorithm to predict residue-residue contacts. Our algorithm explicitly takes into account the occurrence of direct vs indirect dependencies by using all columns in the multiple sequence alignment simultaneously as independent variables to predict the variation in a given column. Existing methods use various approaches such as applying cutoffs based on randomized alignments to distinguish direct from indirect dependencies but RMRCM uses a more principled approach here.

In comparison with MI, we found on simulated datasets that our approach has a better performance in predicting network edges. Note that our simulation model might be somewhat limited in its ability to reflect biological reality but we used it here as an initial test for our method. Analysis of CASP and PFAM cases indicates a very good performance of our algorithm in cases where enough sequences were available. When analyzing additional biological datasets, we found in most of these a comparable or better distance enrichment for RMRCM compared to existing algorithms, in combination with much higher numbers of predicted links by RMRCM. Also, a combination of MI and our new approach seems particular powerful. A clear dependence of RMRCM contact prediction performance on the number of sequences in the alignment was observed, such that until at least ~1,000 sequences performance increases when adding more sequences. Nevertheless, also with sequence alignments with less sequences RMRCM predictions can be competitive compared to existing algorithms for correlated mutation analysis. Although currently the requirement of ~1,000 homologous sequences for a given protein of interest is still somewhat restrictive, one would expect that for proteins that are present in a large enough range of species quite soon the current explosion in sequence data due to the ongoing revolution in sequencing technology will alleviate that restraint. Of particular relevance here are ongoing projects to sequence hundreds or even thousands of different species (http://genome10k.soe.ucsc.edu/, http://solgenomics.net/organism/sol100/view, http://www.bgisequence.com/eu/scientific-initiatives/projects/1000-plants-and-animals/) For alignments with too few sequences, RMRCM would probably not be the method of choice for predicting residue contacts based on sequence data only.

One additional feature that we plan to add to RMRCM is to take amino acid similarity (based on e.g. BLOSUM) into account, by using a prior and/or penalty term that forces amino acids in a given response-column that are similar to each other (e.g. K and R) to obtain links with amino acids in each predictor-column that are similar to each other (e.g. D and E), or that promote contacts between "complementary" amino acids (e.g. K or R with D or E). We expect that this might further boost performance. Also, currently we do not correct for the effect of phylogenetic relationships in the sequence alignment that we use as input [12,37]; doing so might further improve RMRCM.

During the preparation of this manuscript, a novel method, GREMLIN, appeared which also uses regularization to learn a graphical model structure based on sequence data [38]. In several computational aspects, including the exact formulation of the regularization, RMRCM is different from GREMLIN. More important, however, is that the focus of that study was on validating the approach by calculating the imputation error, i.e. the probability of not being able to generate a complete sequence given an incomplete one. As such, that study, and the current study where we focus on residue contact prediction and interaction prediction, are complementary.

An interesting and useful aspect of RMRCM is that it is possible to choose subsets of residue interactions to be taken into account. In particular, we tested using either only intermolecular contacts or both intra- and intermolecular contacts; note that existing pairwise correlated mutation approaches *per se *analyze intermolecular contacts separately from intramolecular contacts in the sense that they analyze pairs of columns, where each pair is either intramolecular or intermolecular. Hence, predictions for intramolecular contacts do not influence predictions for intermolecular contacts, or the other way around. In RMRCM this is different, because we can choose to use either all positions in the alignment as explanatory variables or just a subset. For the prediction of residue contacts, although we did not find much difference for the SK-RR dataset, for the PDZ-peptide dataset the use of both intra- and intermolecular links in the model gave much better results for intermolecular contact prediction compared to using only intermolecular links in the model. For the prediction of *C. elegans *PDZ-peptide interactions based on human interaction data, the situation was reverse: a model trained using only intermolecular links performed better. This might be because the *C. elegans *PDZ sequence similarity to human PDZ sequences overwhelms any intermolecular contribution to the scoring of a *C. elegans *PDZ - peptide pair when including intramolecular links.

## Conclusions

To conclude, the validation using simulated data as well as biological data, demonstrates the usefulness of RMRCM. We believe RMRCM is a versatile framework which will prove quite useful in the annotation of protein sequences.

## Authors' contributions

JS implemented the algorithm, carried out part of the validation and application studies, and drafted the manuscript. CJFtB participated in the design of the study and the implementation of the algorithm. RCHJvH participated in the design of the study and helped drafting the manuscript. ADJvD conceived of the study, participated in its design and coordination, carried out part of the validation and application studies, and helped to draft the manuscript. All authors read and approved the final manuscript.
